# Decreased hypertrophic differentiation accompanies enhanced matrix formation in co-cultures of outer meniscus cells with bone marrow mesenchymal stromal cells

**DOI:** 10.1186/ar3889

**Published:** 2012-06-22

**Authors:** David JJ Saliken, Aillette Mulet-Sierra, Nadr M Jomha, Adetola B Adesida

**Affiliations:** 1 Laboratory of Stem Cell Biology and Orthopaedic Tissue Engineering, Department of Surgery, Division of Orthopaedic Surgery, University of Alberta, Edmonton, AB, T6G 2R3, Canada

## Abstract

**Introduction:**

The main objective of this study was to determine whether meniscus cells from the outer (MCO) and inner (MCI) regions of the meniscus interact similarly to or differently with mesenchymal stromal stem cells (MSCs). Previous study had shown that co-culture of meniscus cells with bone marrow-derived MSCs result in enhanced matrix formation relative to mono-cultures of meniscus cells and MSCs. However, the study did not examine if cells from the different regions of the meniscus interacted similarly to or differently with MSCs.

**Methods:**

Human menisci were harvested from four patients undergoing total knee replacements. Tissue from the outer and inner regions represented pieces taken from one third and two thirds of the radial distance of the meniscus, respectively. Meniscus cells were released from the menisci after collagenase treatment. Bone marrow MSCs were obtained from the iliac crest of two patients after plastic adherence and *in vitro *culture until passage 2. Primary meniscus cells from the outer (MCO) or inner (MCI) regions of the meniscus were co-cultured with MSCs in three-dimensional (3D) pellet cultures at 1:3 ratio, respectively, for 3 weeks in the presence of serum-free chondrogenic medium containing TGF-β1. Mono-cultures of MCO, MCI and MSCs served as experimental control groups. The tissue formed after 3 weeks was assessed biochemically, histochemically and by quantitative RT-PCR.

**Results:**

Co-culture of inner (MCI) or outer (MCO) meniscus cells with MSCs resulted in neo-tissue with increased (up to 2.2-fold) proteoglycan (GAG) matrix content relative to tissues formed from mono-cultures of MSCs, MCI and MCO. Co-cultures of MCI or MCO with MSCs produced the same amount of matrix in the tissue formed. However, the expression level of aggrecan was highest in mono-cultures of MSCs but similar in the other four groups. The DNA content of the tissues from co-cultured cells was not statistically different from tissues formed from mono-cultures of MSCs, MCI and MCO. The expression of collagen I (*COL1A2*) mRNA increased in co-cultured cells relative to mono-cultures of MCO and MCI but not compared to MSC mono-cultures. Collagen II (*COL2A1*) mRNA expression increased significantly in co-cultures of both MCO and MCI with MSCs compared to their own controls (mono-cultures of MCO and MCI respectively) but only the co-cultures of MCO:MSCs were significantly increased compared to MSC control mono-cultures. Increased collagen II protein expression was visible by collagen II immuno-histochemistry. The mRNA expression level of *Sox9 *was similar in all pellet cultures. The expression of collagen × (*COL10A1*) mRNA was 2-fold higher in co-cultures of MCI:MSCs relative to co-cultures of MCO:MSCs. Additionally, other hypertrophic genes, MMP-13 and Indian Hedgehog (*IHh*), were highly expressed by 4-fold and 18-fold, respectively, in co-cultures of MCI:MSCs relative to co-cultures of MCO:MSCs.

**Conclusions:**

Co-culture of primary MCI or MCO with MSCs resulted in enhanced matrix formation. MCI and MCO increased matrix formation similarly after co-culture with MSCs. However, MCO was more potent than MCI in suppressing hypertrophic differentiation of MSCs. These findings suggest that meniscus cells from the outer-vascular regions of the meniscus can be supplemented with MSCs in order to engineer functional grafts to reconstruct inner-avascular meniscus.

## Introduction

The meniscus of the knee joint serves a variety of critical functions that include shock absorption, cartilage protection, and joint stability [1-3]. The capacity to perform these functions is by virtue of its extracellular matrix (ECM) composition and assembly, which is accomplished entirely by meniscus fibrochondrocytes [4,5]. The ECM consists predominantly of type I collagen throughout, type II collagen in the inner meniscus, and proteoglycans, of which aggrecan is predominant [6-8]. Unfortunately, the reparative capacity of the meniscus is hindered by limited vascularization [9]. In human meniscus, the capillary plexus supplies only the outer one third [10] whereas the inner two thirds are avascular; if left untreated, defects in this portion do not heal and may lead to further joint degeneration [11,12]. Current treatment options include partial and total meniscectomies, depending on the extent of meniscal injury [13]. However, these procedures are major risk factors for the early development of osteoarthritis (OA) [13-16]. Cell-based regenerative medicine and tissue engineering have been advocated options to produce functional substitutes to aid repair or replace damaged tissue [17-28]. However, current protocols suffer from several drawbacks that include insufficient numbers of differentiated meniscus cells and loss of ECM-forming phenotype of *in vitro*-propagated meniscus cells. Therefore, alternative cell sources or cell-based strategies are of interest in meniscus tissue engineering.

Adult-derived mesenchymal stromal stem cells (MSCs) are of particular interest in meniscus tissue engineering because of their capacity to undergo differentiation into a variety of mesenchymal lineages, including cartilage and bone [17,29,30]. Additionally, MSCs secrete soluble trophic factors that are capable of promoting cell proliferation and differentiation and suppressing local immune system through both autocrine and paracrine mechanisms [31-35]. However, specific factors known to induce MSCs toward the meniscus cell phenotype are unknown. Co-culture of MSCs with primary meniscus cells is a stratagem that both provides inductive factors and mitigates the need for meniscus cell expansion and associated dedifferentiation of meniscus cells during *in vitro *culture expansion [32,34-38]. Recent work in our laboratory demonstrated that supplementation of human primary meniscus cells with human bone marrow MSCs results in increased meniscus matrix-forming phenotype [39]. Similar work by Cui and colleagues [40] additionally demonstrated that co-cultures of primary human meniscus cells with bone marrow MSCs result in suppression of hypertrophic differentiation of bone marrow MSCs. However, both studies used meniscus cells taken from the whole meniscus and it is not clear whether the cells from the outer and inner menisci will interact similarly or distinctly with bone marrow MSCs. Previous studies have shown that the meniscus cells from the outer and inner regions of the meniscus have distinct ECM gene profiles as well as different multi-lineage differentiation characteristics [41-43]. Moreover, inner meniscus cells display a more chondrogenic phenotype relative to outer meniscus cells on the basis of inherently higher collagen II and aggrecan content [42,43]. Furthermore, outer meniscus cells displayed a more chondrogenic response to hypoxic stimuli by enhanced expression of chondrogenic genes, collagen II and Sry-related HMG box 9 (sox9), than cells isolated from the inner and avascular regions of the meniscus [44]. In this study, we explored the concepts that (a) cells of outer and inner menisci have the capacity to enhance matrix formation of meniscus cells after co-culture with MSCs and (b) cells from the outer and self-healing regions of the meniscus may serve as a clinical source of cells in co-culture tissue engineering strategies with MSCs to recapitulate the functional matrix-forming phenotype of the inner meniscus, which has a limited reparative capacity.

## Materials and methods

### Collection of bone marrow specimens and culture of bone marrow stem cells

Bone marrow aspirates were obtained from surgically discarded material after approval and a waiver of informed consent of the local ethical committee of the University of Alberta (Edmonton, AB, Canada) during routine orthopedic procedures from the iliac crest of two female donors (ages of 23 and 59 years). The number of nucleated cells in the aspirates was determined by crystal violet nuclei staining and counting on a hemocytometer. Thereafter, 15 million nucleated cells per 150-cm^2 ^tissue culture flask were seeded. Culture medium was alpha minimum essential medium supplemented with 10% heat-inactivated fetal bovine serum, 1 mM sodium pyruvate, 100 mM HEPES buffer, 1 mM sodium pyruvate, 100 U/mL penicillin, 100 μg/mL streptomycin, and 0.29 mg/mL L-glutamine (all from Invitrogen, Mississauga, ON, Canada) and 5 ng/mL of basic fibroblast growth factor 2 (FGF-2) (Humanzyme-Medicorp Inc., Montreal, QC, Canada). Nucleated cells were allowed to adhere and grow for 7 days before the first media change under normal oxygen tension (21% O_2 _and 95% air) at 37°C in a humidified incubator with 5% CO_2_. Thereafter, media change was implemented twice per week until 70% to 80% cell confluence was attained. These adherent bone marrow MSCs were detached by using trypsin-ethylenediaminetetraacetic acid (trypsin-EDTA) (0.05% wt/vol) and expanded until passage 2 prior to experimental use. We included FGF-2 throughout MSC cultivation as it maintains human MSCs in an immature state for *in vitro *expansion [45].

### Isolation of human meniscus cells

Menisci were obtained from surgically discarded material after approval and a waiver of informed consent of the local ethical committee of the University of Alberta. Both lateral and medial menisci were harvested from the knee joint of four female donors (age of 55 to 71 years and mean age of 62 ± 6.8 years) undergoing total knee arthroplasty. Tissue from the outer and inner regions represented pieces taken from one third and two thirds of the radial distance, respectively. Meniscus cells were released via treatment with type II collagenase (0.2% wt/vol; Worthington, Lakewood, NJ, USA) after 16-hour digestion of tissue at 37°C in a standard medium-high glucose Dulbecco's modified Eagle's medium containing 4.5 mg/mL D-Glucose (DMEM-HG), 0.1 mM non-essential amino acids, 1 mM sodium pyruvate, 100 mM HEPES buffer, 1 mM sodium pyruvate, 100 U/mL penicillin, 100 μg/mL streptomycin, and 0.29 mg/mL L-glutamine (all from Invitrogen) supplemented with 10% fetal bovine serum (Invitrogen). Meniscus cells from the outer meniscus region were labeled as MCO, and those from the inner portion of the meniscus were designated as MCI. The cell suspension obtained after digestion was passed through a 70-μm nylon-mesh filter. Isolated cells were plated at 104 cells/cm2 and cultured in standard medium for 24 hours under normal oxygen tension (21% O2 and 95% air) at 37°C in a humidified incubator with 5% CO2 before experimental use. Non-adherent cells were aspirated off while adherent cells were detached with trypsin-EDTA (0.05% wt/vol) (Invitrogen).

### Chondrogenic differentiation

Mono-culture in pellets of MSCs, MCO, and MCI were formed in 250 μL of serum-free chondrogenic culture medium consisting of standard medium supplemented with 0.1 mM ascorbic acid 2-phosphate, 10^−5 ^M dexamethasone, 1x ITS+1 premix (Sigma-Aldrich, Oakville, ON, Canada), and 10 ng/mL transforming growth factor-beta 1 (TGF-β1) (Humanzyme-Medicorp Inc.) as described previously [35]. A total of 2.5 × 10^5 ^cells were spun in 1.5 mL of sterile conical polypropylene microfuge tubes (Enzymax LLC, Lexington, KY, USA) at 1,500 revolutions per minute for 3 minutes to form spherical cell pellets. Co-cultures in pellet form consisting of MCO/MSCs or MCI/MSCs were formulated by mixing the two cell types at a 1:3 ratio, respectively. Preliminary studies with a variety of ratios had shown a ratio of 1:3 to reproducibly result in enhanced matrix formation, but identification of the optimal cell-cell ratio remains to be determined [46]. After 3 weeks of culture with media changes three times per week, pellets were processed biochemically for glycosaminoglycan (GAG) and DNA contents, histologically, and immuno-histochemically and by quantitative reverse transcription-polymerase chain reaction for gene expression analysis. For each experiment (four in total), there were two experimental groups (co-culture of MCO/MSCs and co-culture of MCI/MSCs) and three control groups (mono-culture of MSCs, mono-culture of MCO, and mono-culture of MCI). At least two or three replicate pellets per control or experimental group were assessed for each experiment.

### Biochemical analysis

All *in vitro *cultivated pellets were rinsed in phosphate-buffered saline (Invitrogen) and digested in proteinase K (1 mg/mL in 50 mM Tris with 1 mM EDTA, 1 mM iodoacetamide, and 10 mg/mL pepstatin A; all from Sigma-Aldrich) for 16 hours at 56°C. The sulphated GAG content was measured by 1,9 dimethylmethylene blue binding (Sigma-Aldrich) by using chondroitin sulphate (Sigma-Aldrich) as standard [47]. The DNA content was determined by using the CyQuant cell proliferation assay kit (Invitrogen) with supplied bacteriophage λ DNA as standard. Based on experimental GAG values of mono-culture of MSC, MCO, or MCI pellets, the expected GAG values were calculated as a linear function of the proportion (percentage) of MSCs and MCO or MSCs and MCI by using the following equations [35,39]:

$$\text{GA}{\text{G}}_{\text{expected}}=\left[\left(\text{GA}{\text{G}}_{\text{1}00\%\text{ MSCs}}\times \%\text{ MSCs}\right)+\;\left(\text{GA}{\text{G}}_{\text{1}00\%\text{ MCI}}\times \%\text{ MCI}\right)\right]$$

$$\text{GA}{\text{G}}_{\text{expected}}=\left[\left(\text{GA}{\text{G}}_{\text{1}00\%\text{ MSCs}}\times \%\text{ MSCs}\right)+\left(\text{GA}{\text{G}}_{\text{1}00\%\text{ MCO}}\times \%\text{ MCO}\right)\right].$$

The interaction index or measure of chondro-induction was then calculated as the ratio of measured GAG (that is, GAG_measured_) in co-cultured pellets to expected GAG (that is, GAG_expected_). If the value of the ratio is greater than 1, then chondro-induction, an enhanced chondrogenic matrix formation, is considered to have taken place [35,39].

### Histology and immuno-histochemistry

Tissues generated from the pellet cultures were fixed in 4% phosphate-buffered formalin, processed into paraffin wax, sectioned at 5 μm, and stained with 1% Alcian blue and counterstained with 1% neutral red stain to reveal sulphated proteoglycan (GAG) matrix depositions. Other sections were probed with antibodies raised against collagen types I and II. Sections were digested with trypsin and then incubated with antibodies against collagen I (MAB3391) from Millipore (Temecula, CA, USA) or collagen II (II-II6B3) from the Developmental Studies Hybridoma Bank at the University of Iowa (Iowa City, IA, USA). Immuno-localized antigens were visualized with goat anti-mouse IgG biotinylated secondary antibody (Dako Canada Inc., Mississauga, ON, Canada) and a streptavidin-horseradish peroxidase labeling kit with 3,3'-diaminobenzidine (Dako Canada Inc.). Images were captured by using an Omano OM159T biological trinocular microscope (Microscope Store, Roanoke, VA, USA) fitted with an Optixcam summit series 5MP digital camera and Optixcam software and assembled in Adobe Photoshop (Adobe Systems Inc., Mountain View, CA, USA).

### Gene expression analysis

Total RNA was extracted from pellets by using Tri-Reagent (Sigma-Aldrich) after grinding with Molecular Grinding Resin (Geno Technology Inc., St. Louis, MO, USA) in combination with the use of an Aurum Total RNA Fatty and Fibrous Tissue Kit (Bio-Rad, Mississauga, ON, Canada) and after removal of contaminating genomic DNA from the pellets by DNase treatment. To minimize changes in gene expression during cell pellet harvest, cell pellets were immediately (less than 1 minute) transferred into Tri-Reagent. Total RNA (100 ng) in a 40-μL reaction was reverse-transcribed to cDNA by using GoScript reverse transcriptase primed with oligo(dT)_15 _primer (Fisher Scientific, Whitby, ON, Canada). Quantitative real-time polymerase chain reaction was performed in DNA Engine Opticon II Continuous Fluorescence Detection System (Bio-Rad) by using hot start Taq and SYBR Green detection (Eurogentec North America Inc., San Diego, CA, USA). Primer sequences for collagen I (*COL1A2*), collagen II (*COL2A1*), collagen × (*COL10A1*), aggrecan, versican, SOX9, and β-actin (Table 1) were taken from previously published work or were custom designed by using Primer Express software (Applied Biosystems, Foster City, CA, USA) [44,48,49]. All primers were obtained from Invitrogen. Gene (mRNA) expression levels for each primer set were normalized to the expression level of β-actin by the 2-^Δct ^method [50].

**Table 1:** Primer sequences used in quantitative real-time polymerase chain reaction

Gene	Primer	Direction	Reference or GenBank accession number
*Beta-actin *	**5'-AAGCCACCCCACTTCTCTCTAA-3'** *5'-AATGCTATCACCTCCCCTGTGT-3'*	*Forward* *Reverse*	[36]
Aggrecan	*5'-AGGGCGAGTGGAATGATGTT-3'* *5'-GGTGGCTGTGCCCTTTTTAC-3'*	*Forward* *Reverse*	[36]
Collagen I (COL1A2)	*5'-TTGCCCAAAGTTGTCCTCTTCT-3'* *5'-AGCTTCTGTGGAACCATGGAA-3'*	*Forward* *Reverse*	[36]
*Collagen II* (COL2A1*)*	*5'-CTGCAAAATAAAATCTCGGTGTTCT-3'* *5'-GGGCATTTGACTCACACCAGT-3'*	*Forward* *Reverse*	[36]
*Collagen × (*COL10A1*)*	*5'-CAAGGCACCATCTCCAGGAA-3'* *5'-AAAGGGTATTTGTGGCAGCATATT-3'*	*Forward* *Reverse*	[35]
IHh	5'-CCTTGTCAGCCGTGAGGCCG-3' *5'-GCTGCCGGCTCCGTGTGATT-3'*	*Forward* *Reverse*	NM_00218.3
MMP-13	5'-TGGAAGGATGCCTTTTTTTCTC-3' *5'-CACCCTCCCCAAGTATCAATAGG-3'*	*Forward* *Reverse*	NM_002427
SOX9	*5'-CTTTGGTTTGTGTTCGTGTTTTG-3'* *5'-AGAGAAAGAAAAAGGGAAAGGTAAGTTT-3'*	*Forward* *Reverse*	[31]
*Versican*	*5'-*TGGAATGATGTTCCCTGCAA-3' *5'-*AAGGTCTTGGCATTTTCTACAACAG-3'	*Forward* *Reverse*	[35]

All primers were purchased from Invitrogen (Mississauga, ON, Canada). COL1A2, type II collagen α2 chain; COL2A1, type I collagen α1 chain; COL10A1, type × collagen α1 chain; IHh, Indian Hedgehog; MMP-13, matrix metalloproteinase 13; SOX9, Sry-related HMG box 9.

### Statistical analysis

Data are presented as mean ± standard error of mean of measurements. All statistical analyses were performed by using SPSS version 18 (PASW Statistics 18; SPSS Inc., Chicago, IL, USA) unless stated otherwise. Experimental and control groups were compared with one-way analysis of variance with Tukey's multiple comparison *post hoc *tests. Statistical differences between chondro-induction indices of MCO and MSC experimental groups and/or MCI and MSC experimental groups were assessed by paired two-tailed Student *t *test distribution. All statistical differences were considered to be significant with a *P *value of less than 0.05.

## Results

### Enhanced synthesis of glycosaminoglycan matrix in co-cultured pellets of meniscus cells and mesenchymal stromal cells

**Figure 1:**
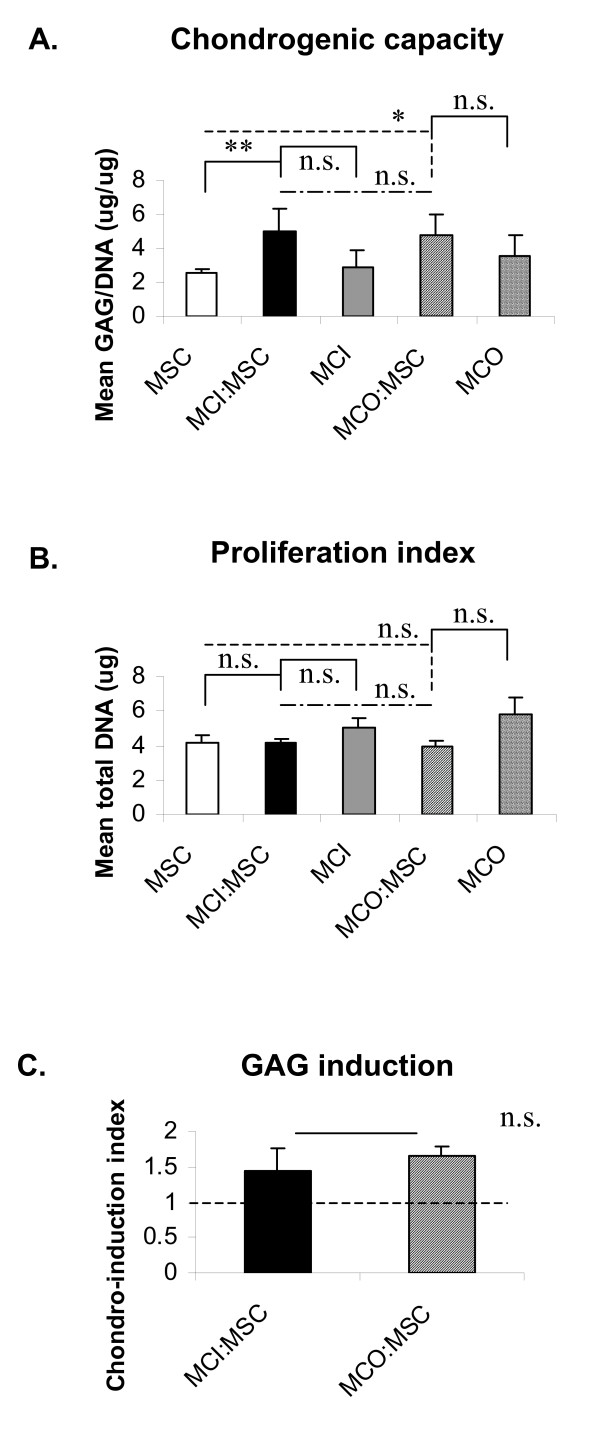
**Chondrogenic capacities of outer or inner meniscus cells in co-culture with mesenchymal stromal cells (MSCs) are similar**. Biochemical analysis was used to evaluate the chondrogenic differentiation capacities (GAG/DNA) and the chondrogenic differentiation indices of the pellets from MSCs, MCI/MSCs, MCI, MCO/MSCs, and MCO after 3 weeks of culture in chondrogenic medium (transforming growth factor-beta 1, dexamethasone, and ascorbate). Chondrogenic induction index was calculated with equations 1 and 2 in the Biochemical analysis subsection of Materials and methods. If the chondro-induction index was greater than 1, an enhancement in chondrogenic matrix formation was considered to have occurred. Data are presented as mean ± standard error of mean of four donor pairs. One-way analysis of variance with Tukey's multiple comparison post tests: *significance when compared with mono-cultures of meniscus cells - outer meniscus cells (MCO) or inner meniscus cells (MCI) - and MSC pellet controls (*P *<0.05); **significance when compared with MCO or MCI and MSC pellet controls (*P *<0.0001). Paired two-tailed Student *t *statistics: no significance (n.s.) (*P *>0.05) between chondro-induction indices of co-cultures of MCO or MCI and MSCs. GAG, glycosaminoglycan; MCI, meniscus cells from inner meniscus region; MCO, meniscus cells from the outer meniscus region.

To characterize the ECM accumulated after pellet culture, the amount of GAG matrix production per cellular DNA content was quantified (Figure 1A). All pellets from co-cultured cells displayed a significantly higher (*P *<0.01) GAG per DNA content (a measure of chondrogenic capacity) than pellets derived from mono-cultures of MSCs. However, the GAG per DNA content of co-cultured pellets was not significantly (*P *>0.05) different from that of pellets formulated from mono-cultures of MCI or MCO (Figure 1A), thus indicating that the enhanced capacities to synthesize the cartilaginous-type ECM of the MCO or MCI in co-culture with MSCs are similar.

To determine whether changes in the number of cells accounted for the difference or similarities in chondrogenic capacities of the co-cultured pellets relative to pellets from mono-cultures of MSCs, MCO, and MCI, we determined the DNA content since DNA content per cell is 11.6 pg [51]. All pellets were not significantly (*P *>0.05) in DNA content (Figure 1B).

To quantify the different enhanced capacity of the co-cultured pellets and MSCs to produce GAG-type matrix, the chondrogenic index was calculated as the ratio of the experimental GAG value to the expected GAG value. Expected GAG value was determined as a linear function of the proportion of meniscus cells (that is, MCI or MCO) and MSCs in co-cultured pellets (Equations 1 and 2). Chondrogenic indices that exceeded 1 were considered to display chondro-induction, which is an enhancement in chondrogenic capacity. All co-cultured pellets had chondro-induction indices of above 1. Chondro-induction indices ranged from 1.2 to 2.1 for MCI/MSC co-cultures and from 1.4 to 2.2 for MCO/MSC co-cultures (Figure 1C). However, there was no significant difference between the two groups.

### Histological assessment of matrix deposition in co-culture of meniscus cells and mesenchymal stromal stem cells

**Figure 2:**
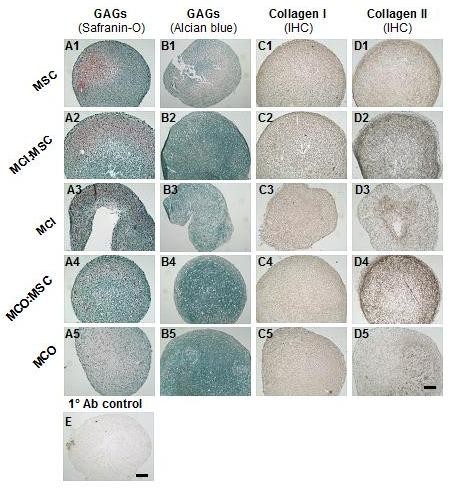
**Histological and immunohistochemical analysis of mono-cultured and co-cultured cell pellets after 21 days of culture**. The expression of matrix proteoglycans (glycosaminoglycanated proteins) in pellet cultures was detected by Safranin O (series A) and Alcian blue/neutral red stain (series B). Extracellular matrix deposition of collagen types I (series C) and II (series D) was detected by immuno-histochemistry (IHC) in mono-cultured (MCI, MCO, and MSC) and co-cultured (MCI/MSCs or MCO/MSCs) cell pellets after 3 weeks of culture in chondrogenic medium (transforming growth factor-beta 1, dexamethasone, and ascorbate). Representative primary antibody control pellets of a 1:3 MCO/MSCs ratio is shown in E. All photomicrographs were captured via a 10× magnification lens. Scale bar is 100 μm. Ab, antibody; MCI, meniscus cells from inner meniscus region; MCO, meniscus cells from the outer meniscus region; MSC, mesenchymal stromal cells.

Cells were cultured from four donor pairs; primary meniscus cells from the outer (MCO) or inner (MCI) region of the meniscus with bone marrow MSCs were cultured, in separate pellets (mono-cultured pellets) or after being mixed (co-cultured pellets), for 3 weeks in serum-free medium supplemented with chondrogenic factors (TGF-β1, ascorbic acid, and dexamethasone). Representative data from co-cultured pairs of four donors are shown in Figure 2. All five groups were positive for proteoglycan staining by safranin O (Figure 1, 2) and Alcian blue (Figure 1, 2) after chondrogenic stimulation (Figure 2). Relative to the mono-cultured pellets (Figure 2B1, B3, and 2B5), the Alcian blue staining intensity in co-cultured pellets (Figure 2B2 and 2B4) appeared to be more intense and homogenously distributed.

Immuno-histochemical assessment of all mono-cultured and co-cultured pellets was performed by using antibodies to types I and II collagen. All pellets stained positively (brown coloration) for types I and II collagen. Representative photomicrographs for collagen I are shown in Figures 1, 2 and for collagen II in Figures 1, 2. Collagen I stain was evenly distributed in all cell pellets (Figures 1, 2). In contrast, with the exception of mono-culture pellets of MSCs (Figure 2D1), collagen II staining was not homogenously distributed over entire pellets (Figure 2). Mono-cultured pellets of MCI (Figure 2D3) were labeled predominantly with collagen II in the central portion of the pellets, whereas pellets from MCO (Figure 2D5) were labeled positively with collagen II in one area of the pellets. Co-cultured pellets of MCI (Figure 2D2) or MCO (Figure 2D4) with MSCs displayed a more intense brown coloration for collagen II than any of the mono-cultured pellets especially prominent at the peripheral rim of the co-cultured pellets. Representative primary antibody control pellets consisting of a 1:3 MCO/MSCs ratio were negative and showed no false background staining (Figure 2E).

### Gene expression profile in mono-cultures and co-cultures of meniscus cells and mesenchymal stromal stem cells

**Figure 3:**
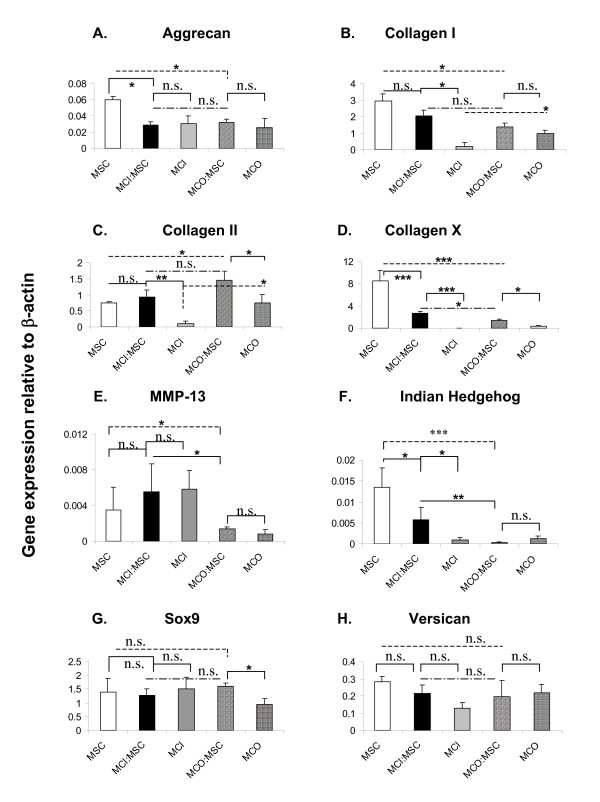
**Gene expression analysis of mono-cultured and co-cultured cell pellets after 21 days of culture**. Quantitative mRNA gene expression analysis via SYBR Green detection was used to evaluate the expression of a panel of fibrogenic (Collagen I and Versican) and chondrogenic (Aggrecan, Collagen II and SOX9) marker genes as well as the expression of genes characteristic of hypertrophic differentiation of MSCs (Collagen X, MMP-13 and Indian Hedgehog) in pellets from MSCs, MCI/MSCs, MCI, MCO/MSCs, and MCO after 3 weeks of culture in chondrogenic medium (transforming growth factor-beta 1, dexamethasone, and ascorbate). Each data point represents the mean ± standard error of the mean of four donor pairs. One-way analysis of variance with Tukey's multiple comparison post tests: *significance when compared with mono-cultures of meniscus cells - outer meniscus cells (MCO) or inner meniscus cells (MCI) - and MSC pellet controls (*P *<0.05) or when compared with mono-cultures of MCO versus mono-cultures of MCI; **significance when compared with co-cultures of MCO or MCI and MSC pellet controls (*P *<0.01); ***significance when compared with MCO or MCI and MSC pellet controls (*P *<0.0001). No significance (n.s.) (*P *>0.05). All marker gene expression was presented as relative mRNA level normalized to human β-actin (y-axis); (A) Aggrecan, (B) Collagen I (*COL1A2*), (C) Collagen II (*COL2A1*), (D) Collagen × (*COL10A1*), (E) MMP-13, (F) Indian Hedgehog (IHh), (G) Sox9, and (H) Versican MCI, meniscus cells from inner meniscus region; MCO, meniscus cells from the outer meniscus region; MMP-13, matrix metalloproteinase 13; MSC, mesenchymal stromal cell; SOX9, Sry-related HMG box 9.

The relative mean expressions of aggrecan, collagen I (*COL1A2*), collagen II (*COL2A1*), collagen × (*COL10A1*), matrix metalloproteinase 13 (*MMP-13*), Indian Hedgehog (*IHh*), *SOX9*, and versican were evaluated at the mRNA level in pellets derived from mono-cultures of MCI, MCO, and MSCs and from co-cultures of MCI or MCO with MSCs (Figure 3). Mono-cultures of MSCs significantly (*P *<0.05) expressed the highest (twofold) level of aggrecan mRNA relative to all other pellet cultures (Figure 3A). There was no statistical difference between aggrecan expression in mono-cultures of MCI or MCO and co-cultures of MCI or MCO with MSCs (Figure 3A).

There was no statistically significant difference between the relative mean expression of *COL1A2 *in co-cultures of MCO or MCI with MSCs (Figure 3B). The expression of *COL1A2 *was significantly higher (approximately 10-fold, *P *<0.0001) in mono-cultures of MSCs and in co-cultures of MCI/MSCs compared with mono-cultures of MCI but was not different between mono-cultures of MSCs and co-cultures of MCI/MSCs (Figure 3B). In contrast, there was no statistical difference between the relative mean expression of *COL1A2 *in mono-cultures of MCO and co-cultures of MCO/MSCs (Figure 3B).

The mean expression of *COL2A1 *mRNA was twofold higher in co-cultured pellets of MCO/MSCs relative to mono-cultures of MSCs/MCO (Figure 3C). The expression of *COL2A1 *in co-cultures of MCI/MSCs was not significantly different (*P *>0.05) from its expression in mono-cultures of MSCs. However, *COL2A1 *expression in co-cultures of MCI/MSCs was ninefold (*P *<0.001) greater than its expression in mono-cultures of MCI (Figure 3C).

The gene expressions of hypertrophic genes, *COL10A1*, *MMP-13*, and *IHh*, were determined (Figure 3D-F). The relative mean expression level of *COL10A1 *was significantly different (*P *<0.001) and highest in mono-cultured pellets of MSCs relative to other mono-cultured and co-cultured pellets (Figure 3D), and its expression was least in mono-cultures of MCI or MCO. The addition of MCI or MCO to MSCs in co-cultured pellets resulted in a highly significant suppression of *COL10A1 *mRNA when compared with its expression in mono-cultures of MSCs (Figure 3D). MCO significantly (*P *<0.05) suppressed the mRNA expression of *COL10A1 *by twofold more than its MCI counterpart (Figure 3D). The relative mean expression level of *MMP-13 *was not statistically different between mono-cultures of MSCs and MCI, nor was there any statistical difference between mono-culture MSCs and co-cultures of MSCs and MCI (Figure 3E). However, the mean relative expression of *MMP-13 *in mono-cultures of MSCs was threefold higher than its expression in co-cultures of MSCs and MCO. The expression of *MMP-13 *was fourfold higher in MSC/MCI co-cultures relative to expression in MCO/MSC co-cultures (Figure 3E). The expression of *IHh *was highest in mono-cultures of MSCs and lowest in co-cultures of MCO and MSCs (Figure 3F). The expression of *IHh *in mono-cultures of MSCs was 2.3-fold higher (*P *<0.05) than in co-cultures of MSCs and MCI. However, when *IHh *gene expression in MSC mono-cultures was compared with its expression in MCO/MSC co-cultures, it was 44-fold higher (*P *<0.0001). Additional comparison showed that the expression of *IHh *in MCI/MSCs relative to expression in MCO/MSCs was 18-fold higher (*P *<0.001) (Figure 3F).

The expression of Sox9, a transcription factor known to facilitate *COL2A1 *expression, was not significantly different between most pellet cultures except in mono-cultures of MCO, where its relative mean expression level was almost twofold lower compared with the other pellets (Figure 3G). There was no statistically significant difference between mono-cultured and co-cultured pellets with regard to mRNA expression of versican (Figure 3H).

## Discussion

Our previous study demonstrated that direct cell-to-cell co-culture of primary human meniscus cells from all regions of the meniscus with bone marrow-derived MSCs resulted in increased matrix formation [39]. However, we did not determine if the cells from the outer-vascular and inner-avascular regions of the meniscus interacted with MSCs similarly or otherwise. The response of cells of outer and inner menisci to culture conditions and growth factors is different, and the expression of *COL2A1 *and aggrecan is higher in monolayer cultures of porcine inner meniscus cells than in outer meniscus cells [42]. We showed that, in aggregate cultures of human inner and outer meniscus cells in the presence of TGF-β1, hypoxia (5%) increased the expression of *Sox9 *in outer meniscus cells but not in inner meniscus cells [44].

In this study, we isolated cells from the inner and outer regions of the meniscus and co-cultured the cells separately with bone marrow-derived MSCs by using the scaffold-free three-dimensional pellet model of tissue formation. Co-culture of primary inner or outer meniscus cells with MSCs resulted in enhanced matrix formation since the calculated interaction indices were greater than 1. This finding was consistent with the data from our recent work on co-culture of whole meniscus cells with MSCs [35]. Furthermore, our results corroborated the findings of similar studies that involved co-culture of chondrocytes or intervertebral discs with MSCs and that resulted in enhanced matrix synthesis [32,35]. The mechanism underlying the enhancement of matrix synthesis was not explored in this study. However, it was interesting to find that the cellularities of the pellets (as judged by the DNA content of our pellets) were not statistically different among experimental and control groups. In a previous study involving co-culture of primary chondrocytes and MSCs, enhanced matrix synthesis was attributed to a synergistic increase in chondrocyte proliferation and induced chondrogenesis of MSCs [35]. However, the lack of difference between the DNA contents of the pellets in this study suggests that the underlying mechanism is most likely independent of changes in cell proliferation. Moreover, our DNA data are consistent with the findings of Furumatsu and colleagues [43], who showed that the proliferation rates of both inner and outer human meniscus cells were the same in the presence of TGF-β1 (10 ng/mL) as used in this study. Other mechanistic possibilities may involve a synergistic cross-talk mediated by soluble factors from both meniscus cells and MSCs [31,52].

Histological and biochemical analyses of the pellets in this study revealed that the cells from the outer and inner regions of the meniscus interacted almost indistinguishably with MSCs. Proteoglycan staining via safranin O and Alcian blue stains were similar, and the GAG content normalized to DNA content were also similar (Figure 1). This finding was surprising as Furumatsu and colleagues [43] recently reported that human inner meniscus cells have a higher GAG content than outer meniscus cells, even after cultivation in chondrogenic factors. The reason for this is not clear but may be associated with differences in the severity of OA in the specimens used in this study. Furumatsu and colleagues used specimens from medial-affected OA joints (that is, specimens from joints with sufficient articular cartilage of lateral femoral condyle and undegenerated lateral meniscus), whereas this study used both lateral and medial menisci from joints with macroscopically diverse, degenerated articular cartilage of lateral and medial femoral condyles. This possibility is further supported by the findings of Sun and colleagues [53], who demonstrated that altered gene expression profiles exist between OA and normal menisci.

Collagen I (*COL1A2*) is the predominant collagen type and ECM molecule in meniscus [5,6]. Co-culture of inner or outer meniscus cells with MSCs resulted in the upregulation of *COL1A2 *transcription, as we previously reported [39]. Similarly to previous findings, at the protein level, collagen I deposition was qualitatively the same in all pellets. Furthermore, there was no statistical difference between its transcriptional expressions in the two co-cultures. Similarly to our previous findings with co-cultures of whole meniscus cells and MSCs [39], the levels of expression of *COL1A2 *in co-cultures of inner or outer meniscus cells with MSCs appear to be orchestrated by the MSC component since the level of *COL1A2 *expression in mono-cultures of MSCs was not surpassed. The underlying mechanism for this regulatory role of MSCs is not clear at this point but may be related to trophic activities of MSCs [31,54]. Nonetheless, our data were consistent with our previous findings that *COL1A2 *expression is higher in human outer meniscus cells than in inner meniscus cells chondrogenic culture [44].

We investigated the gene expression of aggrecan, which is the predominant proteoglycan of the meniscus [55], and versican, another proteoglycan expressed in the meniscus [56], to confirm our histological and biochemical findings. The relative expression levels of aggrecan and versican were not statistically different between co-cultures of inner or outer meniscus cells with MSCs, nor was there any difference between their expression in mono-cultures of inner and outer human meniscus cells (Figure 3A). These findings seem contrary to previous reports that the mRNA expression of aggrecan is higher in inner meniscus cells than in outer meniscus cells. Further contrasts to previous findings were noted in our results on collagen II (*COL2A1*) expression. Our data demonstrated that the expression of *COL2A1 *was least in mono-cultures of inner meniscus cells and highest in co-cultures of outer or inner meniscus cells with MSCs relative to mono-cultures of MSCs or outer meniscus cells. However, previous findings by us [44] and Furumatsu and colleagues [43] demonstrated that *COL2A1 *and *Sox9 *(a transcription factor known to promote *COL2A1*) expression in inner meniscus cells was higher relative to their expression in outer meniscus cells, even in the presence of chondrogenic factors. A plausible reason for this disparity between our findings here and those of previous studies is donor-donor variability (age, gender, and so on) or, as alluded to earlier, the association with possible differences in the severity of OA among study specimens [53,57]. Additionally, it is probable that differences in the multi-potential characteristics of outer and inner meniscus cells play a contributing factor. Mauck and colleagues [41] showed that bovine meniscus cells from the inner and outer regions of the meniscus exhibit different multi-lineage differentiation capabilities. Cells from the outer region were more plastic than inner meniscus cells, differentiating along all three mesenchymal lineages (adipogenesis, chondrogenesis, and osteogenesis). Mauck and colleagues also showed that, when outer or inner meniscus cells were treated with chondrogenic factors (as in this study), inner and outer cell populations expressed similar levels of aggrecan. In contrast, in the absence of chondrogenic factors, the expression of aggrecan in inner meniscus is significantly higher than its expression in outer meniscus [42]. Despite the dissimilarities in our aggrecan and *COL2A1 *data relative to previous reports, it was interesting that the expression of *Sox9 *and *COL2A1 *correlated linearly in mono-cultures of MSCs and MCO and in co-cultures of MCO/MSCs but inversely in mono-cultures of MCI (Figure 3C,E). The expressions of *Sox9 *and *COL2A1 *often are not correlated [57-59], but our findings suggest that, although *Sox9 *is necessary for *COL2A1 *expression, other factors may facilitate *COL2A1 *transcription in inner meniscus. These factors may be particularly active in specimens derived from OA joints and perhaps reflect the similarities of the inner meniscus and articular cartilage [30,53,57]. The deposition and distribution of collagen II were notably enhanced in co-cultures of inner or outer meniscus cells with MSCs and in mono-cultures of MSCs. This correlated with our previous findings involving whole meniscus cells and MSCs [39].

Further gene expression analysis showed statistically significant differences between the expressions of hypertrophic genes, *COL10A1*, *IHh*, and *MMP-13*, in co-cultures of MSCs with outer or inner meniscus cells. *COL10A1*, *IHh*, and *MMP-13 *are predominantly expressed by hypertrophic chondrocytes during endochondral ossification [60]. *COL10A1 *is also a hypertrophic marker for OA cartilage and OA meniscus [61,62]. Expression of both *IHh *and *MMP-13 *modulates hypertrophic differentiation of chondrocytes during endochondral ossification. *IHh *is produced by hypertrophic chondrocytes and, in turn, stimulates secretion of parathyroid hormone-related protein (PTHrP), which acts directly on the chondrocytes to slow the pace of hypertrophic chondrocyte differentiation during endochondral ossification [63]. Increased MMP-13 expression and activity have been implicated in extensive reorganization of ECM during endochondral ossification [62]. Our results showed that outer meniscus cells had a greater potency to suppress hypertrophic differentiation of MSCs than inner meniscus cells. This finding was particularly surprising since inner meniscus cells have been shown to display a reduced osteogenic plasticity relative to outer meniscus cells [41]. The reason for this difference is unclear, but it is plausible that outer meniscus cells have a greater capacity to secrete PTHrP than inner meniscus cells. PTHrP was shown to suppress the mRNA expression of *COL10A1 *[64]. This aspect of the study merits further investigation.

Co-culture of outer or inner meniscus cells with MSCs exhibit similar capacity to synthesis cartilaginous matrix with increased collagen II and proteoglycan content relative to mono-cultures of MSCs, outer or inner meniscus cells. This finding suggests that primary meniscus cells from the outer and reparative region of the meniscus, after supplementation with MSCs, can in principle be used to generate functional grafts or substitutes for the reconstruction of irreparable or degenerate inner meniscus. This possibility is supported by the findings of Kobayashi and colleagues [65], who showed that explants of the outer meniscus integrated well after transplantation to the inner-avascular region of the meniscus. Additionally, our finding regarding the enhanced potency of outer meniscus cells (compared with inner meniscus cells) to suppress hypertrophic differentiation of MSCs makes it an attractive cell source for the reconstruction of the inner meniscus. Furthermore, supplementation of primary meniscus cells with MSCs will mitigate the need to expand meniscus cells for cell-based meniscus tissue engineering purposes since expanded meniscus cells lose their functional matrix-forming phenotype during *in vitro *expansion.

## Conclusions

Taken together, our results demonstrate that outer or inner meniscus cells interact similarly and synergistically with MSCs to increase matrix formation in the presence of chondrogenic factors. However, outer meniscus cells showed a more potent capacity to suppress the expression of hypertrophic differentiation of MSCs than cells from the inner region of the meniscus. These results suggest that the combination of outer meniscus cells and MSCs can be used in cell-based tissue engineering strategies to reconstruct the inner meniscus.

## Abbreviations

COL1A2: type II collagen α2 chain; COL2A1: type I collagen α1 chain; COL10A1: type × collagen α1 chain; ECM: extracellular matrix; EDTA: ethylenediaminetetraacetic acid; FGF-2: basic fibroblast growth factor 2; GAG: glycosaminoglycan; MCI: meniscus cells from inner meniscus region; MCO: meniscus cells from the outer meniscus region; MMP-13: matrix metalloproteinase 13; MSC: mesenchymal stromal stem cells; OA: osteoarthritis; PTHrP: parathyroid hormone-related peptide; SOX9: Sry-related HMG box 9; TGF-β1: transforming growth factor-beta 1.

## Competing interests

The authors declare that they have no competing interests.

## Authors' contributions

DJJS helped to perform experiments and data acquisition and shared responsibility for tissue procurement. AM-S helped to perform experiments and data acquisition. NMJ shared responsibility for tissue procurement, data analysis, and manuscript writing. ABA conceived and designed the study, performed experiments and data acquisition and analysis, wrote the manuscript, and supervised the entire study. All authors read and approved the final manuscript.
